# Factors Associated With Adherence to Outpatient Follow-Up in Patients With Idiopathic Intracranial Hypertension (IIH)

**DOI:** 10.3389/fopht.2021.770807

**Published:** 2021-10-22

**Authors:** Rem Aziz, Asha Shah, Heather E. Moss

**Affiliations:** ^1^ Faculty of Medicine, University of British Columbia, Vancouver, BC, Canada; ^2^ Faculty of Medicine, Cambridge Health Alliance, Cambridge, MA, United States; ^3^ Departments of Ophthalmology and Neurology & Neurological Sciences, Stanford University, Palo Alto, CA, United States

**Keywords:** idiopathic intracranial hypertension, outpatient care, resource utilization, follow-up compliance, sociodemographic factors, appointment adherence

## Abstract

Idiopathic intracranial hypertension (IIH) is a chronic condition characterized by raised intracranial pressure of undetectable origin, that causes morbidity due to debilitating headaches and vision loss. Continuity of outpatient care is important to monitor for permanent vision loss, manage symptoms and limit emergency care. The purpose of this retrospective study was to identify factors associated with neuro-ophthalmology follow-up appointment completion among patients with IIH at a US academic medical center in order to establish evidence-based interventions to improve adherence patterns. Included are 111 completed or no-show neuro-ophthalmology return outpatient appointments by 23 subjects with IIH. Generalized estimating equation models were used to assess association between appointment completion status and factors previously shown to be associated with appointment adherence. Appointments were more likely to be completed during the summer (p=0.08) and by subjects with headache symptoms (p=0.06), however none of the patient factors reached statistical significance. Completed and no-show appointments did not differ by subject demographic or insurance factors. Further studies are needed to identify risk factors for lack of appointment adherence by patients with IIH, particularly those amenable to intervention, in order to improve continuity of care for IIH.

## Introduction

Idiopathic intracranial hypertension (IIH) is a disorder of increased intracranial pressure (ICP), classically occurring in overweight women of childbearing age (1). As per its name, the cause of IIH is unknown, and diagnosis requires confirmation of lack of secondary causes for high ICP such as brain tumor, cerebral venous sinus thrombosis and meningitis. Heterogeneity of IIH is seen with a wide range of visual outcomes including disease progression in some patients despite treatment compliance, thus necessitating escalation of therapy and surgical intervention in some cases (2–4). Furthermore, IIH is a chronic condition that can worsen following periods of stability, thereby warranting longitudinal follow-up to optimize outcomes (5). This highlights the importance of ongoing longitudinal follow-up in preventing progression and sudden deterioration in visual outcomes.

Successful longitudinal care requires adherence to ongoing outpatient appointments and the ability to predictably identify those at risk of being lost to follow up. To establish evidence-based interventions that improve follow-up adherence, it is necessary to first identify factors associated with it. A host of internal and external factors influence adherence behavior, and thus shape health outcomes in return. These can be organized into five major domains: socioeconomic factors, health system-related factors, therapy-related factors, condition-related factors, and finally, patient-related factors (6). Accordingly, the goal of the present study was to identify factors associated with completion of recommended outpatient follow-up appointments by IIH patients.

## Methods

This is a retrospective study of adult patients with IIH followed by an urban academic neuro-ophthalmology service in the University of Illinois Hospital & Health Sciences System (UIH). This project was reviewed by the University of Illinois at Chicago (UIC) institutional review board under expedited procedures with a waiver of informed consent and waiver of HIPAA authorization.

Subjects were identified by chart review. Adult subjects (≥21 years old) with confirmed IIH diagnosis, who completed at least one outpatient neuro-ophthalmology appointment between June to September 2014, were included. For these subjects, all neuro-ophthalmology follow-up encounters between 2012-2015 were abstracted. Canceled, rescheduled, and patient-initiated encounters were excluded. The reason for these exclusions was to focus on patient adherence to provider recommended follow-up. The included encounters were classified as completed or no show.

Subject demographic factors including age, race/ethnicity, zip code, insurance status (private or government) were abstracted from the medical record. Residential distance from the clinic was calculated through Google Maps, using the subject’s zip code to measure the shortest driving distance from home to UIH. Health status was determined based on prescription of medications for diseases other than IIH.

Appointment factors were abstracted from the electronic scheduling system and medical record, and included: time of day (morning or afternoon), season (winter, spring, summer, fall), follow-up time from last completed appointment (1 week, 1 month, 2 months, 3 months, 6 months), whether subject is receiving treatment for IIH, IIH medication change at or since prior appointment, and symptoms at last appointment (headache or not).

Appointment status was the primary outcome. Generalized Estimating Equation (GEE) analysis was applied to model this as a dichotomous outcome (no show *vs*. completed) using logit link with subject as a grouping variable to account for within subject correlations. Unadjusted analysis was performed for each considered subject and appointment factor. Analysis was performed using SPSS 26 (IBM Inc).

## Results

Twenty-three subjects with IIH (mean age 39, all female, 14 (60%) Black non-Hispanic, 0-66 miles between residence and clinic, 19 (82%) on IIH medication, 11 (47%) with Medicaid insurance) had 321 appointments scheduled between 2012-2015. Two hundred and ten were excluded for being cancelled, rescheduled, initial consultation, patient-initiated, visual field only or undocumented appointments. The remaining 111 appointments were categorized as completed (n=92, 83%, range 1-12 per patient) or no-show (n=19, 17%, range 0-5 per patient) visits.

None of the patient factors were associated with adherence to outpatient appointments. Appointments scheduled in the summer season had higher adherence, and those with 1 month follow-up interval had lower adherence, but these did not reach statistical significance. Subjects with active headache had better follow-up, but this did not reach statistical significance. There was no association between other appointment factors (time of day, season, follow-up time, treatment status, mediation changes, symptoms reported) and outpatient appointment adherence among IIH patients (**Table 1**).

**Table 1:** Distribution of patient and appointment factors by attendance, and association with non-adherence.

Factors	% No-show (n = 19)	% Completed (n = 92)	Odds Ratio (95% CI)*	P-value*
Age in years (Mean ± SD)	41.7 ± 8.6	43.0 ± 10.6	0.99 (0.94-1.04)	0.64
Race/ethnicity:			1.48 (0.43-5.10)	0.53
Black-non-Hispanic	63%	72%		
Distance from clinic (miles)	0-66	0-23	1.032 (0.98-1.09)	0.28
Insurance status: Public aid	26.3%	32.6%	1.36 (0.61-3.00)	0.46
Health status: Receiving meds	94.7%	84.6%	0.31 (0.06-1.65)	0.17
Time of day: AM	68%	66%	0.91 (0.32-2.55)	0.89
Season				
Spring	26%	19%	1.14 (0.32-4.02)	0.84
Summer	16%	33%	3.16 (0.86-11.60)	0.08
Fall	26%	27%	1.58 (0.49-5.06)	0.44
Winter	32%	21%	ref	
Follow-up time				
1-2 weeks	5%	12%	ref	0.06
1 month	42%	25%	0.26 (0.06-1.08)	0.40
2 months	11%	10%	0.41 (0.05-3.23)	0.73
3-4 months	21%	35%	0.73 (0.12-4.55)	0.33
6 months	21%	18%	0.39 (0.06-2.62)	
IIH treatment: Yes	79%	72%	0.68 (0.18-2.61)	0.57
Recent IIH med change: Yes	47%	39%	0.71 (0.38-1.51)	0.38
IIH symptoms (headache): Yes	53%	76%	2.82 (0.96-8.30)	0.06

*Odds ratio and p-values calculated using GEE models accounting for within subject correlation.

## Discussion

The understanding of factors associated with continuity of outpatient care for those with IIH is important for designing interventions to improve outcomes in this chronic disease. Secondary benefits would include more cost-effective care and reduced overall resource utilization (7). Towards this end, we present a study assessing factors related to outpatient follow-up adherence in patients with IIH, receiving care at an urban academic medical center in the United States.

Our study of established IIH patients in a neuro-ophthalmology practice did not identify intervenable factors associated with outpatient follow-up completion. Trends for seasonal effects existed, with compliance being better in the summer than the winter, possibly attributable to the weather in Chicago or fewer work/school scheduling conflicts. Follow-up completion was better in subjects with persistent symptoms. However, neither of these factors reached statistical significance, perhaps related to sample size. Ongoing study is critical given the importance of outpatient follow-up to allow management of chronic conditions. In other diseases correlations between outpatient adherence and cultural, sociodemographic and psychosocial factors have been demonstrated (8, 9).

Further research is needed to identify patient‐related and health‐system factors that are associated with follow-up care in IIH so that patients at risk of poor follow-up can be identified and appropriately managed. As an example of what might be accomplished, one study evaluating follow-up patterns in HIV-seropositive patients led to proposed interventions to minimize sociodemographic barriers to appointment adherence in order to promote optimal management of disease progression (8). Similarly, literature surrounding diabetes follow-up asserts the influence of psychosocial factors in chronic disease management, highlighting that patients with attachment styles characterized by low levels of collaboration were more likely to miss appointments than those with secure attachment styles (10). It is worth noting that many of those with poor follow-up compliance will naturally be the most challenging to access during observational research studies. Thus, it is important to critically evaluate solutions drawn from studies that focus on more accessible populations, and contextualize them in terms of those most lost to care (11, 12). Future research regarding follow-up disparities in IIH patients will need to be more encompassing of underrepresented populations with greater likelihood of experiencing the greatest health needs.

Intentional *versus* unintentional nonadherence would be another notable avenue to assess as it has shown importance in research concerning compliance of glaucoma patients with ocular hypotensive therapies (13). Additionally, identifying factors affecting adherence in patients from different genders and cultural backgrounds may be significant to understanding follow-up patterns in patients with IIH, majority of whom are females. In a recent study, beliefs about glaucoma treatment were predictive of adherence in patients of western cultures, suggesting possible improvement of adherence patterns through examination of patient concerns and illness perceptions (14). Finally, a thorough assessment of tailored interventions and their impact on medical adherence and health outcomes would strengthen their implementation to help patients with IIH achieve better follow-up.

Our study is based on the assumption that longer follow-up leads to better health outcomes. Though this has not been formally evaluated in patients with IIH, a recent prospective study demonstrated an association between changes in IIH care prompted by the COVID-19 lockdown and changes in management (15). Moreover, established outpatient care has been associated with better outcomes in other chronic disease conditions. In a study of outpatient diabetes management, consistent appointment attendance was a strong predictor of successful disease control (16). Similarly, a case study examining attendance at outpatient clinics identified appointment nonadherence as a significant disadvantage in the provision of effective medical care (17). A literature review looking at nonadherence in chronic disease conditions identified numerous consequences in medical care provision impacting individual patients and overall health systems (18). These included increased wait times, strained patient–provider relationships, and increased system costs in addition to accumulative negative impacts on patient health, which limit continuity of care and disrupt management plans (19–21). Such conditions delay necessary treatment, leaving patients at an increased vulnerability for the development of serious medical complications. Rigorous study is needed to demonstrate the importance of outpatient specialty care in improving IIH outcomes while reducing costs.

Our findings are not without limitations. Generalizability of our study is limited by its retrospective nature and restriction to a single center, thereby excluding geographic variability and possible relevant care obtained from outside providers. We had a small sample size which was driven in part by restriction to subjects with a confirmed IIH diagnosis only. Future work could include a larger sample size to reveal statistically significant associations and enable subgroup analyses. We were unable to capture cultural, sociodemographic, and psychosocial factors, which as discussed earlier, pose important considerations for adherence and follow-up. Future studies may consider directly exploring reasons for poor adherence through the use of patient questionnaires or interviews. Additionally, it would be worthwhile to revisit patients with cancelled and rescheduled appointments, who were originally excluded in our inclusion criteria, to assess whether adherence to follow-up care took place.

In conclusion, our pilot study did not identify specific factors upon which to intervene to establish outpatient care and maintain appointment compliance. Thus, further research is required to better understand factors associated with continuity of care for patients with IIH in order to facilitate a shift to longitudinal outpatient care. Ultimately, this would serve the purpose of continued follow-up for optimal management of IIH, as well as the potential to minimize IIH-related healthcare costs. One particularly interesting approach would be to map out the sequence and stages of care in patients with IIH, such as diagnosis, management and maintenance, in order to identify patterns, their possible causes and appropriate interventions to implement.

## Data Availability Statement

The raw data supporting the conclusions of this article will be made available by the authors, without undue reservation.

## Ethics Statement

The studies involving human participants were reviewed and approved by University of Illinois at Chicago (UIC) institutional review board. Written informed consent for participation was not required for this study in accordance with the national legislation and the institutional requirements.

## Author Contributions

Conceptualization, RA and HM. Methodology, AS, and HM. Formal analysis, AS and HM. Investigation, AS and HM. Resources, HM. Data curation, AS. Writing—original draft preparation, RA. Writing—review and editing, RA and HM. Visualization, RA, AS, and HM. Supervision, HM. Project administration, HM. All authors contributed to the article and approved the submitted version.

## Funding

This research was funded by National Institutes of Health, grant number P30 EY 026877, the National Center for Advancing Translational Sciences, National Institutes of Health, through Grant UL1TR002003 and a Research to Prevent Blindness unrestricted grant to Stanford Department of Ophthalmology.

## Author Disclaimer

The content is solely the responsibility of the authors and does not necessarily represent the official views of the National Institutes of Health.

## Conflict of Interest

The authors declare that the research was conducted in the absence of any commercial or financial relationships that could be construed as a potential conflict of interest.

## Publisher’s Note

All claims expressed in this article are solely those of the authors and do not necessarily represent those of their affiliated organizations, or those of the publisher, the editors and the reviewers. Any product that may be evaluated in this article, or claim that may be made by its manufacturer, is not guaranteed or endorsed by the publisher.
